# Connecting the Dots: Milestones in the History of Extracellular Vesicle Research

**DOI:** 10.3390/ijms27052470

**Published:** 2026-03-07

**Authors:** Joanna Guzowska, Szymon Kowalski, Iga Schachta, Natalia Piekuś-Słomka, Artur Słomka

**Affiliations:** 1 Department of Pathophysiology, Nicolaus Copernicus University in Toruń, Ludwik Rydygier Collegium Medicum, 85-094 Bydgoszcz, Poland; joanna.murawska@doktorant.umk.pl (J.G.); iga.schachta@cm.umk.pl (I.S.); 2 Department of Inorganic and Analytical Chemistry, Nicolaus Copernicus University in Toruń, Ludwik Rydygier Collegium Medicum in Bydgoszcz, 85-089 Bydgoszcz, Poland; natalia.piekus@cm.umk.pl; 3 Department of Hematology and Oncology, National Medical Institute of the Ministry of Interior and Administration, 02-507 Warsaw, Poland

**Keywords:** extracellular vesicles, history

## Abstract

The field of extracellular vesicle (EV) research offers a compelling example of a biological concept refined through continuous methodological innovation. This review traces the historical trajectory of the discipline chronologically, beginning with early observations in haemostasis, from Malpighi’s descriptions of blood clots and Chargaff and West’s identification of a procoagulant sedimentable plasma fraction, to Wolf’s “platelet dust,” Crawford’s microparticles characterised by electron microscopy, and the seminal work by Stahl and Johnstone demonstrating regulated vesicle biogenesis during reticulocyte maturation via multivesicular bodies. We highlight a pivotal conceptual shift, from viewing EVs as cellular debris to recognising them as regulated “communicasomes,” catalysed by Raposo’s discovery of antigen-presenting exosomes and subsequent evidence for EV-mediated transfer of functional receptors and nucleic acids, including the influential and sometimes debated model proposed by Ratajczak. By integrating findings from matrix vesicles, plant-derived vesicles, and diverse tissue contexts, we frame EV release as an evolutionarily conserved process with profound implications for immunity, regeneration, oncology, and cardiovascular pathology. A second central aim of this review is practical and methodological. We map how the expansion of biological claims has driven urgent standardisation efforts, notably through the establishment of the International Society for Extracellular Vesicles (ISEV) and the successive MISEV guidelines (2014, 2018, 2023). These are complemented by community resources such as EV-TRACK, MIFlowCyt-EV, and the databases ExoCarta and Vesiclepedia. We summarise core experimental choices across isolation and characterisation techniques, including ultracentrifugation, size exclusion chromatography, density gradients, flow cytometry, nanoparticle tracking analysis, and electron microscopy, while outlining persistent bottlenecks in purity, standardised nomenclature, and experimental reproducibility. Finally, we provide concise biographical sketches of key contributors and an overview of major EV-focused journals and ISEV meetings that anchor consensus-building and the translation of fundamental knowledge into clinical and industrial applications.

## 1. Conceptual Framework and Definitions

Extracellular vesicles (EVs) constitute a heterogeneous ensemble of membrane-delimited, quasi spherical particles, typically spanning approximately 30 to 1000 nanometers in diameter. Encased within a lipid bilayer, EVs package a complex molecular cargo, including proteins, lipids, and nucleic acids, and may also display functionally active proteins on their surface. Among these constituents, RNA species, including messenger RNA (mRNA), have received particular scrutiny because they can mediate intercellular information transfer by delivering bioactive molecules to recipient cells [1]. EVs are released by a wide spectrum of cell types and have been detected across tissues as well as in numerous body fluids, including clinically accessible biofluids such as blood and urine. Despite long-standing inconsistencies in terminology (e.g., matrix vesicles, exosome-like vesicles, dexosomes), EVs are most commonly classified into three major categories, microvesicles, exosomes, and apoptotic bodies, based on biogenesis and selected physical characteristics [2,3,4,5,6]. Within this framework, EV serves as the umbrella term, whereas microvesicles and exosomes denote distinct subtypes that arise from different formation and release pathways.

Microvesicles (MVs) originate through outward budding and fission of the plasma membrane, a process promoted by cellular activation, stress signalling, and perturbations in the extracellular milieu. Typically measuring approximately 100 to 1000 nm, MVs carry diverse biomolecules, including proteins, lipids, and nucleic acids (notably RNA). By contrast, exosomes are smaller vesicles (approximately 30 to 150 nm) generated within the endosomal system: inward budding of the endosomal membrane produces intraluminal vesicles within multivesicular bodies (MVBs), which subsequently fuse with the plasma membrane to release exosomes into the extracellular space. Apoptotic bodies emerge during apoptosis (programmed cell death), when membrane blebbing and cellular fragmentation produce membrane-enclosed vesicles that can incorporate cytosolic constituents, organelles, and signalling mediators, with potential consequences for neighbouring cells and immune regulation [7,8,9,10].

Collectively, EVs should be viewed not as a single uniform entity, but as a continuum of subtypes and subpopulations that differ in biogenesis, morphology, and molecular composition. These differences underpin functional diversity and enable the selective delivery of cargo to recipient cells. EV secretion has been documented across diverse organisms (nearly all living organisms on Earth), supporting the concept that EV-mediated communication is evolutionarily conserved. In this context, EVs have been termed “communicasomes”, nanoscale, extracellular, organelle-like structures implicated in intra- and intercellular signalling. Under physiological conditions, EVs contribute to intercellular communication, biomolecule transport, immune modulation, and the orchestration of regenerative processes [11]. In pathological contexts, EVs have been implicated in tumour progression, neurodegenerative mechanisms, and cardiovascular disease, and they may propagate immune and inflammatory activation [12,13]. Importantly, EV release is an actively regulated cellular process; accordingly, EVs should not be dismissed as inert cellular debris [14,15,16].

Early descriptions of EV-like structures appeared decades ago; however, systematic progress accelerated from the 1970s onward, and the vast majority of EV research has been conducted within the past two decades. Methodological innovation and advances in analytical technologies now allow investigators to interrogate mechanisms and causal links that were previously experimentally inaccessible, connecting these nanostructures to complex networks of extracellular communication [17,18]. The annual number of EV-related publications has risen sharply since 2010 [19]. One major focus in contemporary EV research concerns their roles in cancer biology, including tumour initiation and metastatic dissemination. Accumulating evidence indicates that cancer cell-derived EVs can reshape the tumour microenvironment and may promote distant metastasis through systemic signalling circuits [12,13,14,15,16,17,18,19,20,21,22]. In parallel, EVs are being developed as therapeutic and biotechnological platforms, including their use as drug delivery vehicles, tools in skin regeneration, and highly specific vaccine scaffolds [23,24,25]. As the field expands and experimental pipelines diversify, community-driven standards and reporting guidelines have become increasingly important, outlining best practices for EV isolation, characterisation, and data disclosure, thereby strengthening interpretability and reproducibility across studies [26].

In this review, we provide a historical perspective on pivotal milestones in EV research and highlight seminal discoveries that shaped current conceptual frameworks. We also present biographical profiles of scientists who made particularly influential contributions to the field. Distinct from conventional topic-centred reviews, this manuscript is intentionally structured as a chronological narrative that exposes how ideas evolved, how definitions sharpened, and how methodological breakthroughs repeatedly forced conceptual reappraisal. By following the discipline across decades, we aim to make the logic of the field legible not only to specialists, but also to readers approaching EV biology from adjacent areas, for whom the story of discovery can be as informative as the technical details. Subsequent sections address outstanding challenges, emerging research directions, and practical considerations for conducting EV experiments with maximal robustness and reproducibility. Finally, we summarise the most recent MISEV recommendations (Minimal Information for Studies of Extracellular Vesicles) and provide an overview of EV-focused journals, as well as major international conferences.

## 2. Tracing the Roots of Extracellular Vesicle Research: A Historical Perspective

Haemostasis-related observations provided the initial entry point into EV research. Since then, the field has matured into a coherent biological framework, and the major conceptual transitions are summarised below.

The origins of EV research can be traced to early efforts to understand blood composition and the mechanisms governing haemostasis. As early as 1666, Marcello Malpighi compared cardiac thrombi with clots formed ex vivo and noted a fibrous, mesh-like organisation, an observation that helped catalyse mechanistic inquiry into coagulation and haemostatic control. In retrospect, this trajectory proved relevant not only for haemostasis research, but also for later attempts to interpret biologically active, sedimentable plasma fractions that would eventually be situated within the EV spectrum [27].

A decisive advance in the twentieth century came through the work of Erwin Chargaff and Randolph West, who were among the first to report that a particulate fraction obtained from blood plasma by high-speed centrifugation exhibits strong procoagulant activity. These particles, later interpreted as vesicular material and now considered part of the broader EV spectrum, prompted further investigations into the biological significance of noncellular, sedimentable plasma components [28,29,30,31].

Erwin Chargaff (1905–2002), best known for Chargaff’s rules of DNA base composition, made influential contributions across the medical sciences beyond nucleic acids. Born in Czernowitz (now Chernivtsi, Ukraine), he studied chemistry at the University of Vienna and subsequently worked, among others, at the Pasteur Institute in Paris and later at Columbia University in New York, where he remained scientifically active for decades. He received numerous distinctions over his career, including the Pasteur Medal (1949), the Carl Neuberg Medal (1958), the Gregor Mendel Medal (1968), and the H.P. Heineken Prize (1973) [28,29]. He died on 20 June 2002 in New York at the age of 96. Importantly in the present context, his coagulation-oriented analyses redirected attention to cell-free, sedimentable plasma material as a plausible locus of biological activity, an idea that, viewed through a modern lens, anticipates the later recognition of vesicle-like structures in blood as functional entities rather than incidental by-products.

Randolph West (1890–1949), Chargaff’s co-author on the seminal coagulation study, was an American haematologist and professor of medicine at the College of Physicians and Surgeons (Columbia University). His research centred on haematological disease and coagulation, and he also contributed to early clinical work related to the “anti-anaemia factor” from liver preparations. In 1948, he reported in *Science*, a leading scientific journal, therapeutic activity of vitamin B_12_ in pernicious anaemia (Addison–Biermer disease) [30,32,33]. Randolph West died unexpectedly in 1949 and was posthumously honoured for contributions to clinical nutrition [34,35,36,37,38,39,40].

In their coagulation experiments, Chargaff and West showed that a plasma fraction sedimenting at high centrifugal force exhibited pronounced procoagulant activity, and they speculated that small particles, likely derived from blood cells, could contribute materially to clot formation [30,31]. Although the tools available at the time did not allow precise structural definition, their observations provided a conceptual bridge from “particulate fractions” in plasma to later, more explicit descriptions of vesicular entities. Building on this, fasting blood was anticoagulated with sodium oxalate, plasma was sequentially centrifuged (including a prolonged high-speed spin), and coagulation was evaluated using a recalcification assay; the authors also conducted plasma mixing studies, supplemented samples with a purified thromboplastic protein preparation derived from bovine lung, and applied protamine (salmine) titration primarily to assess heparin-like anticoagulant activity. Methodological constraints included the absence of ultrastructural imaging and molecular phenotyping, limited standardisation of preanalytical handling, and a consequent risk that the high-speed pellet was heterogeneous (for example, platelet-derived material and cellular debris rather than a uniform vesicle population); accordingly, from a contemporary extracellular vesicle standpoint, these findings are best interpreted as an early functional indication of a sedimentable procoagulant fraction, while modern investigations require standardised, multi-platform characterisation and explicit separation of vesicles bearing tissue factor (TF) from those whose procoagulant activity is driven predominantly by externalised phosphatidylserine (PS).

Subsequent decades refined these observations into concepts that can now be placed within the EV framework. In 1967, Peter Wolf analysed platelet-free plasma and identified a procoagulant particulate material that was neither intact cells nor soluble metabolites. He termed this material “platelet dust”. He then demonstrated that ultracentrifugation could operationally separate this activity from the fluid phase. The supernatant lost thrombin-generating capacity, whereas resuspension of the pellet restored it, indicating that the sedimented particles carried a functionally indispensable procoagulant activity [41,42,43].

In methodological terms, Wolf examined platelet-rich and platelet-free fractions prepared from blood anticoagulated with trisodium citrate or EDTA and recovered a minute sedimentable particulate fraction using dilution and high-speed centrifugation. He supported the interpretation with electron microscopy and quantified procoagulant activity chiefly through PF3-related functional readouts, including thrombin generation and Stypven recalcified clotting assays. Despite representing a methodological step forward relative to earlier particulate-fraction studies, the work still shared similar core limitations, including incomplete standardisation and reporting of preanalytical handling, residual susceptibility to handling-dependent platelet activation, and uncertainty regarding compositional heterogeneity of the sediment, while relying predominantly on morphology and functional assays without marker-based EV profiling, which is now considered essential for robust EV identification and mechanistic attribution, including separation of TF-driven and PS-driven activity.

Importantly, although this material was not yet interpreted as a defined vesicular population and could easily be dismissed as a by-product of platelet handling, Wolf’s experiments established a crucial principle: the procoagulant phenotype of plasma could reside in a discrete, cell-free, sedimentable particulate fraction rather than exclusively in soluble factors. On that basis, he suggested that the pellet contained determinants functionally analogous to tissue factor (TF).

While the 1967 “platelet dust” experiments are frequently highlighted as a conceptual inflexion because they anchored a clinically meaningful coagulation readout in a separable particulate entity, Wolf’s trajectory toward this conclusion began earlier.

Notably, the interpretability of these early particulate-fraction studies, including Wolf’s work, remained constrained by broadly similar preanalytical and compositional uncertainties, which modern EV methodology explicitly aims to control.

In 1956, using density gradient ultracentrifugation, he had already detected platelet-associated activity within small particles that sedimented below the major protein fractions of a human antihaemophilic factor (AHF) concentrate. Subsequent electron microscopy observations further strengthened the interpretation that the material was membrane derived: lipid-rich particles were observed that probably originated from so-called platelet osmophilic granules and were released spontaneously during platelet storage, including in the absence of overt coagulation activation. It was therefore proposed that particle release constitutes an element of platelet activation and that the particulate material detected during clot formation represents a structure distinct from platelets themselves, erythrocytes, or chylomicrons [41,42,43]. This earlier experiment is presented here deliberately, because it foreshadows the logic of Wolf’s 1967 [41] work by linking platelet-associated biological activity to a discrete, sedimentable particulate fraction, and therefore should not be treated as a peripheral observation in accounts of his contribution to the EV literature. Read through a modern lens, Wolf’s “platelet dust” can be viewed as an early, function-driven description of platelet-derived EVs, recognised long before the field had the terminology or conceptual vocabulary to place them within an organised EV taxonomy.

A further methodological and conceptual advance followed with Neville Crawford’s work in 1971 [44]. Working with rigorously prepared platelet-free plasma from humans and several animal species, he operationally isolated a small sedimentable particulate fraction by differential centrifugation and ultracentrifugation under citrate- or EDTA-anticoagulated conditions intended to limit ex vivo platelet disruption. Using electron microscopy, Crawford visualised discrete, membrane-delimited particles and applied complementary biochemical and immunological assays indicating a lipid-bearing structure enriched for ATP and platelet contractile proteins, thereby coupling structural visibility to a coherent molecular signature. This ultrastructural evidence was pivotal, because it suggested that the particulate fraction corresponded to organised entities with a recognisable morphology rather than amorphous precipitates or experimental debris. He further strengthened the biological interpretation through functional systems probing clot retraction and platelet enzyme activity, aligning particle isolation with measurable haemostatic effects. From a modern EV methodological perspective, however, the evidentiary framework remained largely operational, relying on sedimentation and morphology in the absence of orthogonal purification, quantitative particle sizing, and marker-defined phenotyping, leaving residual uncertainty around heterogeneity and possible co-sedimentation of non-vesicular material or platelet-injury products. Notably, the term “microparticles” subsequently gained wide currency in the field, even though current EV terminology employs it more selectively. Together, these observations consolidated the emerging view that such particles warrant interpretation as biologically consequential structures rather than incidental artefacts of coagulation related handling [44]. The history of EV research thus illustrates how careful attention to anomalous signals, coupled with methodological innovation, can progressively crystallise a new biological category.

A pivotal moment in EV history is associated with Rose Mamelak Johnstone (1928–2009), a Polish born biochemist who emigrated to Canada and spent her scientific career at McGill University in Montreal where she became a leading figure in biochemistry and a prominent advocate for women in academic science, including serving as Chair of the Department of Biochemistry (1980–1990) [45,46,47,48]. While her early work addressed tumour metabolism and membrane proteins, in the late 1970s she focused on membrane remodelling during reticulocyte maturation. It was then, as she later acknowledged, that she unexpectedly encountered a phenomenon she described as the “Alice in Blunderland effect”. Her studies indicated that these vesicles are present in plasma independently of standard metabolites or cellular fragments, opening a new chapter in the history of EV discoveries. In doing so, she argued that these structures are not merely by-products but can reflect regulated biology, consistent with earlier observations by Chargaff, West, Wolf, and Crawford, who had documented EV-like particles in the context of coagulation and platelet activity, albeit without the conceptual and terminological framework that would later define the EV field [27]. Johnstone used the “Alice” metaphor to underscore the serendipity of recognising an ordered process where one might have assumed artefact or disposal. Conceptually, this episode did not replicate the earlier haemostasis centred work; rather, it complemented it by reframing particulate material as a regulated output of cellular remodelling. In this sense, Wolf’s ultracentrifugation-based functional readout and Crawford’s ultrastructural visualisation established that a discrete particulate fraction existed and carried biological activity, whereas Johnstone’s reticulocyte model provided a mechanistic and developmental context in which vesiculation could be interpreted as a programmed cellular process. Together, these lines of evidence helped consolidate the foundations for today’s understanding of EVs as organised, biologically active mediators of intercellular communication [44]. Johnstone was not the first to see small vesicles, but she was among the first to insist that they have biological meaning. Her reticulocyte work helped reframe EV release as a regulated step in membrane remodelling, not accidental debris. In a personal essay, she recalled how she found her vocation in the words: “Had I deliberately planned this career I could not have chosen better. The truth is that it occurred by default—a process of weeding out what I did not want to do.” [45,46,47,48]. In 1983, reticulocyte maturation became the proving ground for the next phase of EV research through two independent programmes, one led by Johnstone and the other by Philip Stahl.

Johnstone and colleagues operationalised exosomes in the reticulocyte maturation model by maturing sheep reticulocytes in vitro, collecting conditioned medium, and recovering a vesicle-enriched pellet by high-speed ultracentrifugation; they reported selective enrichment of transferrin receptor in this fraction together with acetylcholinesterase activity and ligand-binding activities linked to glucose and nucleoside transport, while cytosolic enzyme signals were low, supporting an active and selective route of membrane protein disposal during maturation. Stahl, using rat reticulocytes generated by phenylhydrazine-induced anaemia, combined quantitative transferrin uptake and surface stripping assays with ultrastructural tracing using electron-dense transferrin conjugates and high-resolution electron microscopy, localising transferrin to multivesicular endosomal compartments that were largely non-lysosomal by cytochemical criteria and documenting events consistent with fusion to the plasma membrane and extracellular release of intraluminal vesicles.

Prof. Stahl is an emeritus professor of Cell Biology and Physiology at Washington University. During his career, he served as Head of the Department of Cell Biology and Physiology and as Director of the Division of Biology and Biomedical Sciences. In 2017, he was honoured by the International Society for Extracellular Vesicles (ISEV) for pioneering research on EVs. He is at present a co-editor of the second edition of the Encyclopaedia of Cell Biology, published in 2022 [49,50,51].

Both scientific groups worked with a model of reticulocyte maturation and were the first to provide unequivocal evidence that cells can actively remove intracellular material in the form of vesicles [52]. Stahl’s team captured by electron microscopy the release of vesicles from multivesicular bodies (MVBs) that fuse with the plasma membrane, establishing a mechanistic framework for exosome biogenesis. In parallel, Johnstone’s group demonstrated that reticulocytes remove unnecessary transferrin receptors via EV secretion, proposing that this represents a programmed membrane reorganisation step required for mature erythrocyte function [52,53]. The results of these studies not only confirmed the existence of EVs as active biological structures but also initiated a new line of research into their role in cellular differentiation and homeostasis.

Under the MISEV guidelines for small EVs, these findings remain foundational yet methodologically constrained because differential centrifugation and morphology do not establish preparation purity, population homogeneity, or definitive endosomal biogenesis; contemporary reporting would additionally require particle concentration and size-distribution metrics, orthogonal purification such as density gradients or size-exclusion chromatography, and marker panels including EV-associated positives together with negative indicators of intracellular contamination.

In 1983, Pan and Johnstone demonstrated by radiolabelling that transferrin receptors are selectively removed from maturing sheep reticulocytes in association with EVs, supporting the view that vesiculation participates in programmed membrane reorganisation rather than passive disposal [53]. In 1991, Johnstone further developed this concept by showing that EVs released during reticulocyte maturation carry selected plasma membrane proteins and activities that are normally lost as the cell matures. A key implication was selectivity: EV release preferentially removes defined membrane components, and different cargoes can show different apparent efficiencies of recovery, consistent with sorting and differential stability rather than random shedding. In historical terms, the significance of these experiments lay less in the fine quantitative details than in the overarching conclusion that EV formation provides a regulated mechanism for membrane protein disposal during differentiation, thereby placing exosomes within a coherent cellular programme rather than at the periphery of cell biology [54,55,56].

For much of the following decade, EVs were still widely viewed as a selective “waste disposal” mechanism. A major conceptual expansion occurred in the mid-1990s, when Maria da Graça Raposo provided direct evidence that EVs can execute a bona fide immunological function rather than merely reflect cellular turnover. In 1996, she demonstrated that B lymphocytes secrete exosomes carrying MHC class II complexes that remain competent for antigen presentation [57]. This result redefined the interpretive frame for EVs: vesicles were no longer only candidates for selective disposal but could be viewed as structured carriers of immune information. In practical terms, her work established a mechanistic bridge between endosomal trafficking pathways and extracellular communication, and it created a clear translational horizon by linking EV biology to antigen presentation and immunotherapy [58].

Raposo and colleagues isolated vesicles from B lymphocyte culture medium by stepwise centrifugation and then purified them further using a sucrose density gradient. They confirmed vesicle-like structures by electron microscopy and showed MHC class II on these vesicles by immunogold labelling, and they used the lack of transferrin receptor signal to argue against strong contamination by plasma-membrane fragments.

Her subsequent research has continued to focus on EV biogenesis and function in immune and disease contexts, reinforcing the centrality of immune cell-derived EVs in contemporary EV biology [59]. This breakthrough attracted the attention of many researchers, including Dr Sebastián Amigorena, who isolated vesicles released by dendritic cells mainly by high-speed ultracentrifugation, characterised them by electron microscopy and by detecting MHC class I and class II together with tetraspanin-related markers, and then tested their activity in vivo in immunisation and tumour models.

His team showed that dendritic cell-derived exosomes, when loaded with tumour peptides, can activate cytotoxic T lymphocytes in vivo and thereby induce tumour elimination or suppress tumour growth in mice [59]. Together, these findings provided a decisive functional turning point for the field: EVs could be positioned as purposive vectors of immune communication with measurable biological outcomes, rather than passive cellular debris.

In light of MISEV and current methodological standards, both approaches should be described as enrichment workflows rather than proof of a pure, single vesicle population or definite endosomal origin. Neither study reports particle concentration and size distribution as would be expected today for dosing and comparability, and neither applies a broad marker panel that includes both EV-associated positives and negative controls for intracellular or non-vesicular contamination. From today’s perspective, strong functional claims would also require stricter controls to rule out effects from co-isolated soluble proteins, cytokines, or free peptide, because these can remain in ultracentrifugation-based isolates and can influence immune readouts independently of vesicle delivery.

**Figure 1:**
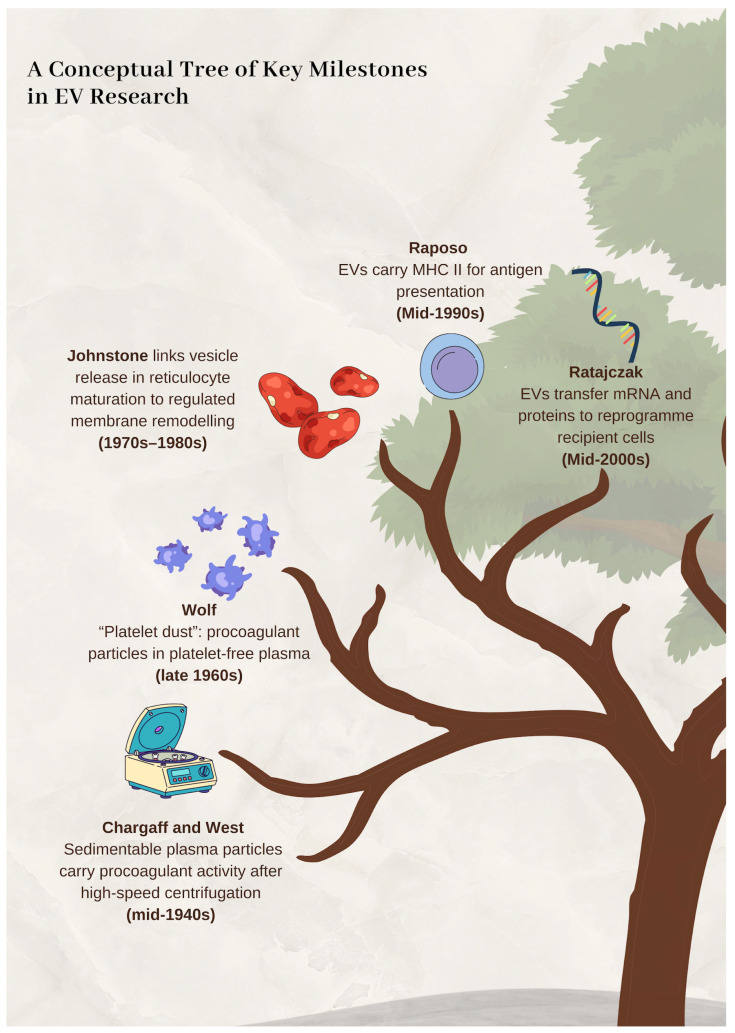
A conceptual tree of key milestones in extracellular vesicle (EV) research. The schematic summarises major conceptual inflexion points that progressively reframed EVs from sedimentable, cell-free particulate activity in plasma to regulated biological entities and, ultimately, to mediators of intercellular information transfer. Approximate time windows are indicated to emphasise the historical sequence rather than exact publication dates; icons illustrate the dominant biological context associated with each milestone (coagulation and plasma fractions, platelet-derived particles, reticulocyte maturation, immune function, and gene-regulatory cargo transfer).

Convergent work in haemostasis, electron microscopy, and mechanistic cell biology progressively displaced the “debris” hypothesis and established EVs as organised biological outputs with defined activities. Importantly, this reappraisal was facilitated by parallel advances in laboratory instrumentation and analytical techniques, which refined the isolation, visualisation, and functional characterisation of EVs. Figure 1 summarises the key landmark steps.

## 3. New Frontiers in EV-Mediated Signalling: The Ratajczak Paradigm Shift

The Ratajczak research programme marked an early, mechanistically explicit reframing of EVs as causal agents rather than incidental correlates. This framework provided a clear, testable conceptual basis that remains influential in contemporary interpretation and is therefore examined here in focused detail.

In 2006, Ratajczak and colleagues presented evidence that EVs released by activated, non-malignant cells can participate in intercellular signalling. They proposed that recipient cells may be stimulated by ligands displayed on the vesicle surface and that EVs may additionally mediate intercellular transfer of adhesion molecules and receptors, thereby altering recipient cell responsiveness. The authors also discussed EV-associated transfer of infectious material, including retroviral particles such as HIV and prions, as a potential mechanism for disseminating bioactive factors between cells [60]. Although the field has since refined terminology and experimental stringency, the conceptual move was decisive: EVs were framed as vehicles capable of propagating biological influence across cellular boundaries.

A second mechanism emphasised in the same research line proved even more consequential, namely horizontal transfer of genetic information. In a widely cited study, Ratajczak’s group reported that stem cell-derived MVs can deliver mRNA and proteins to recipient cells, with measurable effects on cellular phenotype and function [61]. The importance of this observation lies in its mechanistic implication. EVs were no longer only transient extracellular stimuli; they could be interpreted as information-bearing vectors capable of reshaping recipient-cell programmes, at least in defined experimental settings.

Prof. Mariusz Z. Ratajczak received his medical doctorate in Poland and subsequently completed habilitation in internal medicine. His research has focused on stem cell biology, including mechanisms of stem-cell mobilisation and their roles in tissue regeneration. He is also associated with the description of very small embryonic-like stem cells (VSELs), a putative population of rare, very small, quiescent adult tissue cells reported to exhibit a high nucleus-to-cytoplasm ratio and to express selected markers linked to stemness, in adult bone marrow, a finding that stimulated further discussion on adult stem-cell heterogeneity and potential regenerative applications [62,63,64]. Across these themes, his work has repeatedly intersected with EV biology, including analyses of vesicle-associated proteins, lipids, and RNA and their potential impact on haematopoiesis, stem cell trafficking, and cancer-related processes.

Janina Ratajczak is a haematology and stem cell biology researcher and is currently an Assistant Professor in the Department of Medicine at the University of Louisville School of Medicine. She obtained her medical degree from the Pomeranian Medical University in Szczecin (1982), completed specialisation in internal medicine (1990), and defended a PhD on the role of pro-inflammatory cytokines and chemokines in human erythropoiesis in the context of anaemia of chronic disease (1996). Her early clinical and academic work was conducted in Szczecin (including the Rheumatology Department of the Regional Hospital, 1982–1990), followed by postdoctoral training at the University of Pennsylvania (Department of Pathology, 1991–1998). Her current research interests include microRNA-mediated regulation of the cell cycle and insulin signalling in VSELs, as well as the expression and functional significance of chemokine receptors in human megakaryoblasts, topics relevant to understanding stem cell regulation and translational opportunities in regenerative medicine.

In their 2006 work, the authors discussed MVs as an underappreciated class of membrane-derived particles released by both healthy and malignant cells, carrying proteins, lipids, and nucleic acids (including mRNA), as well as membrane receptors; transfer of additional cellular material (including organelles such as mitochondria) was also considered [65]. Within this framework, MVs were proposed to influence recipient cells through three principal routes: (i) ligand-receptor interactions at the vesicle surface, (ii) transfer of surface receptors that can recalibrate signalling competence, and (iii) delivery of intracellular cargo capable of reconfiguring recipient cell programmes [65]. Presented as such, this was not merely a catalogue of vesicle contents, but an attempt to define a mechanistic grammar for EV-mediated communication.

A comprehensive and balanced scholarly assessment necessitates acknowledgment of key scientific controversies that have shaped the discourse surrounding the Ratajczak research programme, as these unresolved questions provide essential context for the field’s development.

The most prominent dispute concerns very small embryonic-like stem cells (VSELs). Specifically, the Weissman laboratory reported an inability to identify adult mouse bone marrow VSELs possessing the claimed stem cell properties. Their study indicated that events within the putative VSEL gate predominantly contained minimal DNA, and the corresponding cells failed to meet key functional and molecular criteria for stem cells. This constituted a failure to confirm the existence of pluripotent VSELs as previously described [66].

Regarding extracellular vesicle (EV)-mediated RNA transfer, experimental evidence presents a nuanced picture. On one hand, reporter-based studies have demonstrated EV uptake and subsequent translation of EV-delivered mRNA cargo in recipient cells [67]. On the other hand, quantitative analyses argue that the transfer of endogenously present EV RNA may be limited under physiological conditions [68]. Furthermore, the precise mechanisms of EV uptake and the ultimate fate of their cargo remain incompletely understood [69].

Methodological challenges also persist, particularly in distinguishing EV subtypes and assigning them distinct functions. The extent to which exosomes differ from other classes of extracellular vesicles in terms of biogenesis and function remains poorly defined [70]. Consequently, the MISEV guidelines explicitly caution against attributing specific biological functions to vesicle preparations that are heterogeneous or insufficiently characterised [71].

Methodological advances underwrote the empirical credibility of these claims. Electron microscopy enabled visualisation of spherical, membrane-enclosed vesicles consistent with active cellular release, while flow cytometry supported phenotypic profiling and source inference based on marker expression; lineage-associated membrane proteins strengthened attribution and linked MV composition to functional hypotheses, including platelet-associated and haematopoietic-associated signatures [65]. Subsequent studies from the same group reported transfer of biologically active cargo, including mRNA, proteins, membrane receptors and, in some contexts, mitochondria, with measurable effects on survival, proliferation and migration of haematopoietic and tumour cells, alongside pro-angiogenic and immunomodulatory remodelling of the tumour microenvironment [72]. Collectively, these findings reinforced EVs as mechanistically interpretable mediators rather than mere biomarkers.

## 4. Morphology Before Mechanism: Early Evidence for MVB-Linked Vesicle Release

This subsection highlights in vivo morphology-first milestones that revealed the cellular architecture of the exosomal route before EV terminology and functional models were established, complementing the haemostasis-centred narrative by pointing to MVB-dependent secretion during a defined physiological transition.

Some of the earliest ultrastructural observations compatible with what is now recognised as the exosomal route can be traced to the mid-1970s. Multivesicular bodies (MVBs) are membrane-enclosed endosomal compartments containing multiple intraluminal vesicles (ILVs). They occupy a central position in endosomal sorting: many MVBs fuse with lysosomes to deliver cargo for degradation, whereas a distinct subset can fuse with the plasma membrane and discharge ILVs into the extracellular space. Once released, these vesicles are commonly termed exosomes. This secretory itinerary is now firmly linked to EV-mediated intercellular communication and has been implicated across diverse physiological settings, including immunity, metabolism, and cellular stress responses [8,73].

In 1974, Eladio Núñez and colleagues described ultrastructural changes in thyroid follicular cells of bats during arousal from hibernation. They reported an increased abundance of vesicle-containing endosomal structures described as resembling MVBs, frequently localised near the apical membrane, alongside other features consistent with heightened secretion. They also noted extracellular vesicles and colloid droplets in this setting, collectively supporting the interpretation of an intensified secretory state during a rapid metabolic transition. Read in hindsight, this study is often cited as an early morphological indication of MVB-associated vesicle release, even though the biological identity and broader relevance of these structures could not be conclusively defined at the time [74].

Núñez’s observations provide ultrastructural localisation of MVB-like compartments adjacent to the secretory membrane and link their dynamics to a defined physiological trigger, arousal from hibernation. Positioned between haemostasis-derived functional observations and later reticulocyte mechanistic proof of ILV release via MVB fusion, they indicate that MVB abundance and positioning are state-dependent and consistent with regulated EV output rather than incidental shedding.

## 5. Beyond the “Platelet Dust”: The Multiple Origins of Extracellular Vesicle Research

Although Peter Wolf’s “platelet dust” and Johnstone’s work on vesicles from maturing red blood cells are key milestones, focusing only on them makes the history of EV research seem like a straight line starting in blood science. This view misses the field’s true, multi-disciplinary beginnings.

In the same year as Wolf’s discovery (1967) [41], plant biologists Halperin and Jensen used microscopes to see plant cells releasing vesicle-like material. They described structures fusing with the cell membrane and releasing their contents [75]. This was an early, independent observation of EV-like activity in plants. This line of research continued in 2009 when Regente and colleagues isolated vesicles from sunflower fluids and linked them to proteins thought to be involved in cell communication [76].

A similar separate path developed in microbiology. Rodrigues and his team showed that a fungal pathogen releases vesicles containing components that help it cause disease. They demonstrated that these vesicles could cross the fungal cell wall [77]. This showed that vesicle release is not just for mammalian cells, but a general biological method for moving complex materials out of cells with tough walls.

Other early studies also took place outside of blood research. In 1969, Anderson found vesicles in cartilage and linked them to the start of bone formation [78]. This gave vesicles a fundamental role in normal development, separate from blood clotting or immunity.

These parallel discoveries demonstrate that research on EVs has always been multidisciplinary. Findings from plant biology, mycology, and bone development were contemporary with work in haematology and immunology. The convergence of these independent lines of inquiry reveals that EVs are not a niche phenomenon. They are a conserved biological mechanism for intercellular transfer present across diverse branches of life.

## 6. Technological Determinants of Conceptual Evolution in Extracellular Vesicle Biology

The progression of EV research from descriptive observation to clinical translation has been contingent upon advances in enabling technologies, with each major conceptual reorientation emerging from expanded analytical capacity.

EV identification began with electron microscopy, enabling visualisation of multivesicular body fusion [75], platelet-derived particles [79], and matrix vesicles [78]. The subsequent recognition of EVs as intercellular communication vectors required enrichment and molecular interrogation: ultracentrifugation enabled discovery of functional RNA shuttling [80], while mass spectrometry established that EV cargo encodes tissue-specific signatures [81], catalysing the shift from “cellular debris” to information-bearing nanoparticles.

Appreciation of EV heterogeneity required single-particle resolution. Nanoparticle tracking analysis exposed quantitative size distributions [82], high-sensitivity flow cytometry enabled subpopulation phenotyping [83], and microfluidic strategies enabled rare-subset isolation from complex biofluids [84]. This resolution proved mechanistically consequential: PD-L1+ EVs exhibit origin-dependent functional heterogeneity, demonstrating that marker positivity alone insufficiently predicts immunosuppressive capacity [85]. Establishing structure–function relationships required integrated multiscale frameworks confirming that isolation methodology directly modulates EV attributes and recipient cell effects [86].

Therapeutic translation remains dependent on prior analytical advances. Loading strategies (electroporation, sonication, chemical conjugation) and surface engineering for targeted delivery [87] rely on proteomically defined target biology, while biomimetic synthetic vesicles combine EV-inspired functionality with synthetic scalability [88]. Cell-type-specific isolation exemplifies persistent technological dependence: solid-tissue-derived EVs constitute <1% of circulating particles, necessitating hybrid affinity-based workflows [89] and bio-orthogonal capture strategies [90].

Over 470 registered clinical trials [91] include diagnostic applications (urine exosome assays for prostate cancer stratification [92]) and therapeutics (KRASG12D-targeting exosomes in phase I pancreatic cancer [93]; dendritic-cell-derived vaccines [94]). Scale-up now determines clinical feasibility: tangential flow filtration demonstrates superior yield versus ultracentrifugation [95]; three-dimensional bioprocessing enhances manufacturing output [96]; cellular nanoporation enables high-yield mRNA-exosome generation [97]. MISEV guidelines provide consensus frameworks for rigour [98], though technical fragilities persist-adsorptive losses and lipoprotein co-isolation distort quantification [89].

The trajectory of EV research demonstrates iterative interdependence between conceptual ambition and technological capacity. Future advances in scalable bioprocessing, quality-control architectures, and definitive clinical efficacy will emerge from continued integration of biological frameworks with enabling technologies.

## 7. Contemporary Consolidation: ISEV and the MISEV Standards

EV research then entered a consolidation phase defined by ISEV-led community building and MISEV consensus guidelines that operationalised standards for rigorous, comparable, and reproducible work in the field.

Founded in 2011, the International Society for Extracellular Vesicles (ISEV) is a non-profit scientific organisation dedicated to advancing EV research through community building, training initiatives, conferences, and the development of field-wide recommendations that promote methodological rigour and reproducibility. Today, ISEV represents a large international community, reported as exceeding 1700 members worldwide, and functions as a central coordinating body for standardisation efforts in EV science. ISEV is governed by elected leadership, and the Society’s presidency has been held by a succession of prominent investigators from different countries: Jan Lötvall (2011–2016), Andrew Hill (2016–2018), Clotilde Théry (2018–2022), Edit Buzás (2022–2024), and Kenneth Witwer (2024–2026).

These presidents are closely associated with major strands of EV research that, collectively, helped shape the field’s trajectory. Jan Lötvall (University of Gothenburg, Sweden) is widely recognised for contributions to EV biology in respiratory disease. Andrew Hill (Victoria University, Australia) has advanced extracellular RNA and EV biomarker research and serves as Editor-in-Chief of the *Journal of Extracellular Biology*. Clotilde Théry (Institut Curie, Paris, France) has been a leading voice in EV biology, including methodological development and consensus-building. Edit Buzás (Semmelweis University, Budapest, Hungary) has investigated EV roles in immune regulation and autoimmune disease. The current president, Kenneth Witwer (Johns Hopkins University, USA), has contributed to EV method development and to rigour and standardisation efforts, including in infectious and oncological contexts.

A major outcome of ISEV’s community activities is the MISEV guideline series, which provides a practical framework for experimental design, EV preparation and characterisation, and transparent reporting. The first document, MISEV2014, represented a milestone by proposing minimal biochemical, biophysical, and functional standards to support robust attribution of EV-associated cargo and functions, with the overarching goal of improving credibility and reproducibility of EV studies [26]. Key aspects of MISEV2014 included a definition of EVs and their classification, recommendations regarding EV isolation and characterisation, and a requirement to provide detailed methodological information in scientific publications.

As knowledge and technologies advanced, an updated and substantially expanded position statement, MISEV2018, was published in 2018 [98]. In contrast to a strict reliance on biogenesis labels, it encouraged terminology anchored in operational descriptors, including physical properties (such as size and density) and biochemical composition. It also sharpened expectations regarding isolation strategies and analytical characterisation, and it explicitly emphasised the need to identify contaminants and potential artefacts. In doing so, MISEV2018 helped convert “best practice” from an informal norm into a shared, field-level reference point, improving comparability across laboratories [71].

The most recent update, MISEV2023, published in 2024, constitutes the next step in this evolving standardisation effort. Its stated goal is to provide an updated snapshot of available approaches, together with advantages and limitations, for production, separation, and characterisation of EVs from multiple sources, including cell culture, body fluids, and solid tissues. MISEV2023 also introduces new sections on EV release and uptake and includes a brief discussion of in vivo approaches; the guideline reflects extensive community input, incorporating feedback from ISEV task forces and more than 1000 researchers.

Collectively, the MISEV series has done more than harmonise reporting. It has reshaped the evidentiary bar in EV research by making transparency, contamination awareness, and cross-study comparability explicit expectations rather than optional refinements. At the same time, it remains a dynamic framework designed to evolve as the field advances. To complement the MISEV framework, ISEV has also built publication and meeting infrastructures that accelerate the circulation of methods, standards, and biological insights across the community. In practice, these outlets have become part of the field’s quality control ecosystem, shaping what is debated, validated, and ultimately accepted as reliable EV evidence.

Over the past decade, critiques of the MISEV initiative have consistently highlighted a fundamental tension: the field requires shared minimum expectations to ensure interpretability and reproducibility, yet any formalised guidance risks being perceived as either excessively prescriptive or insufficiently rigorous, depending on experimental context and epistemic priorities. Community feedback following MISEV2014 revealed this polarisation clearly. While most respondents endorsed the concept of minimal requirements, substantial minorities concurrently judged MISEV as too restrictive or, conversely, not stringent enough, with a smaller subset viewing such requirements as an unwarranted constraint on the field [99]. This polarity persisted in subsequent survey-based analyses informing later updates. Critiques of MISEV2018 clustered around restrictiveness, excessive length, and perceived omissions, alongside concerns that compliance could devolve into superficial box-ticking and that MISEV might be invoked inequitably during peer review [100].

MISEV2018 itself articulates the underlying rationale for why such interpretive variation is difficult to avoid. The evidentiary basis for assigning subtype-specific functions or defining universal markers remains limited, and the biological heterogeneity of extracellular particles renders categorical claims tenable only when supported by rigorous characterisation and appropriate controls. Accordingly, the document cautions that strong claims attributing highly specific biological activities to exosomes remain experimentally difficult to support, given incomplete understanding of biogenesis and overlap with biophysically similar EV populations [71]. In parallel, it states that it is not yet possible to propose universal markers capable of discriminating EV subtypes, including MVB-derived exosomes, across experimental contexts, because available studies rely on divergent isolation approaches and biological sources [71]. These statements serve as an internal justification for why MISEV cannot be reduced to a simple checklist: the underlying measurement problem is underdetermined, and the logic of universal markers is not currently defensible at the level some readers expect.

The nomenclature debate illustrates how methodological uncertainty can propagate into semantic inflation. A focused commentary on terminology argues that the term exosome has undergone definitional dilution, being applied variably to small EVs, all EVs, or poorly characterised cell releasates, frequently without demonstration of the exclusivity the term implies [55]. From a standards perspective, this is not merely a linguistic issue. It describes a systematic pathway through which imprecise labelling facilitates mechanistic and functional claims that are not adequately supported by evidence, a failure mode MISEV seeks to constrain but cannot fully prevent.

A complementary line of criticism holds that the field’s principal bottleneck is not the absence of guidelines, but chronic underreporting of essential experimental parameters. The foundational rationale of EV-TRACK is that EV research requires more transparent reporting to facilitate interpretation and replication, motivating structured reporting infrastructure designed to operationalise best-practice metadata [101]. When reporting quality is quantified via the EV-METRIC, the post-MISEV era shows improvement, although the magnitude remains modest at the population level. Average compliance rose from 19.8% to 24.7% after MISEV, with stronger performance in manuscripts that cite MISEV compared to those that do not [102]. This pattern supports a nuanced interpretation: guidelines can shift norms, but adoption remains heterogeneous and incomplete, perpetuating methodological ambiguity and dissatisfaction among researchers facing resource constraints or reviewer expectations.

By 2023 and 2024, another critique became unavoidable: scale. As EV methods and applications diversified, MISEV expanded in length, topical breadth, and operational detail, increasing the risk that a document intended as minimal information would be perceived as a prescriptive governance instrument. MISEV2023 therefore pre-empts recurrent misreadings by stating explicitly that it is not a one-size-fits-all blueprint and by cautioning that the guidance should not stifle innovation. It emphasises fit-for-purpose decision-making rather than mandatory procedural uniformity [98]. This positioning aligns with a later perspective on how and when MISEV should be updated, which notes that updates have taken progressively longer to write and publish, and that the reports have grown considerably in length, scope, and detail, thereby increasing coordination costs and extending the update cycle [103]. The implicit structural critique is that once a guideline becomes a large consensus artefact, revision becomes a multi-year enterprise, potentially creating a lag between methodological innovation and codified recommendations [103].

Finally, perhaps the most consequential critique emerges at the translational interface. Even as expectations for isolation and characterisation are increasingly articulated in the research literature, clinical trial records frequently fail to disclose these parameters. A critical review of EV clinical trials reports that only 12.1% of study records specified an isolation technique and only 36.1% identified any EV characterisation method, describing this under-specification as standing in stark contrast to the research community’s broader push for reporting and standardisation [91]. This gap is not merely administrative. Without methodological transparency, inter-trial comparability is undermined, and the evidentiary chain linking an EV product or biomarker signal to its claimed biological substrate becomes difficult to audit [91].

Taken together, the verified literature indicates that criticism of MISEV is best understood as a family of critiques targeting different layers of the same underlying problem. Some critiques address perceived stringency and feasibility constraints across heterogeneous laboratory settings [104]. Others target sociotechnical misuse, whereby recommendations may be treated as mandates in peer review or reduced to performative compliance [100]. Another set addresses epistemic limits, namely the lack of universal subtype markers and the fragility of strong functional claims [71]. Yet another critiques nomenclature inflation and its downstream impact on mechanistic inference [55]. Finally, multiple sources converge on a persistent reporting deficit that guidelines alone cannot resolve, motivating complementary infrastructure and exposing a translational transparency gap in clinical trial documentation [91,101,105].

Beyond guidelines, ISEV supports dissemination of EV research through dedicated Gold Open Access journals. The Society’s flagship journal, the *Journal of Extracellular Vesicles* (JEV), was launched in 2012 as the official ISEV journal and has since served as a key venue for EV biology, methodology, and consensus papers [101]. The journal enables the exchange of data, ideas, and information on the chemistry, biology, and applications of EVs. Articles cover signal transduction, cellular uptake and dissemination of EVs, EV based pharmacological, immunological, and genetic approaches, as well as cellular mechanisms, including membrane transport and protein sorting, associated with vesicle release and EV biogenesis. The Editors-in-Chief are Jennifer Jones, Hang Hubert Yin, and Pascale Zimmermann, supported by Deputy Editors, Associate Editors, and reviewers from the EV community [102]. A second ISEV journal, the *Journal of Extracellular Biology* (JEB), was established in 2021 to broaden the publication scope across EVs and related extracellular particles and processes, spanning mechanisms of biogenesis and transport to EV-mediated intercellular communication in physiology and disease. Andrew Hill serves as Editor-in-Chief, supported by an international editorial team [106,107]. ISEV also organises the annual flagship conference, the ISEV Annual Meeting (Table 1), which covers a broad range of EV biology, medicine, and technology and provides a central forum for exchange of methods and emerging findings. Notably, due to the COVID-19 pandemic, ISEV2020 and ISEV2021 were held in virtual formats.

Together, these journals and meetings operationalise standardisation by giving the field shared venues in which methods can be stress-tested, consensus statements can be iterated, and new claims can be evaluated against community expectations. In that sense, ISEV has not only supported EV research, but has helped define the practical conditions under which it can progress with rigour and reproducibility [108,109].

**Figure 2:**
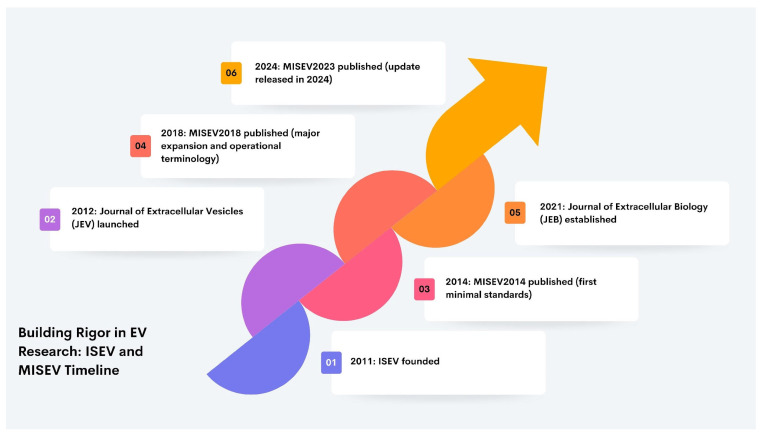
Key milestones in contemporary EV field consolidation (2011–2024). The schematic highlights six pivotal developments that accelerated community coordination and standardisation in EV research: the founding of the International Society for Extracellular Vesicles (ISEV) in 2011; the launch of the *Journal of Extracellular Vesicles* (JEV) in 2012; publication of MISEV2014 as the first minimal framework for EV study design, characterisation, and reporting; the expanded MISEV2018 update introducing more operational terminology and strengthened expectations for methodological transparency; establishment of the *Journal of Extracellular Biology* (JEB) in 2021; and publication of the MISEV2023 update released in 2024, reflecting an updated consensus on approaches to EV production, separation, and characterisation across multiple sources.

As a leading international society in the EV field, ISEV supports research standardisation, steers the discipline through consensus-driven priorities, and convenes regular meetings that showcase methodological and conceptual advances. Its membership has expanded steadily and now comprises nearly 2000 researchers worldwide. Through its journals, annual meetings, and continued institutional growth, the Society has helped consolidate field-wide expectations for rigour, transparency, and reproducibility in EV science [107]. Figure 2 provides a concise visual chronology of the major ISEV- and MISEV-linked developments that institutionalised standardisation and strengthened rigour in EV research between 2011 and 2024.

Beyond the MISEV series, several community resources further reinforce transparent reporting, data sharing, and reproducible practice. EV-TRACK provides a structured reporting checklist and methodology knowledgebase that facilitates critical appraisal and cross-study comparability [110]. For flow cytometry-based studies, MIFlowCyt-EV offers a minimum-information framework specifically tailored to EV detection, analytical workflow disclosure, and reporting standards in this technically sensitive area [83]. In parallel, curated databases such as Vesiclepedia and ExoCarta enable systematic exploration of EV cargo across organisms and experimental contexts, supporting hypothesis generation and contextualisation of new findings within the broader literature [111,112].

Complementing community repositories such as Vesiclepedia and ExoCarta, ISEV has established Special Interest Groups (SIGs), i.e., member-driven networks that concentrate expertise around defined EV functions or biological contexts. SIGs are intended to intensify interactions and knowledge exchange within focused thematic areas, thereby providing a structured environment that can catalyse discoveries, methodological and technological advances, and research talent development through specialised meetings, educational activities, and continuous communication of key advances. Operationally, SIGs are coordinated by up to four expert (co-)chairs drawn from all three ISEV geographical chapters (Europe and Africa, Americas, and Asia-Pacific), supported by broader expert boards; participation is open to ISEV members via enrolment through the society’s member portal. At present, ISEV lists two established SIGs, EVs in Nervous System (EViNS) and Genitourinary System EVs (GUSEV), exemplifying how the society organises topic-specific communities and activities to move distinct EV subfields forward.

The narrative pivots from discovery to discipline building, outlining how EV research became verifiable at scale. ISEV and the successive MISEV iterations (2014, 2018, 2023), reinforced by journals, annual meetings and resources such as EV-TRACK, MIFlowCyt-EV, Vesiclepedia and ExoCarta, operationalised rigorous expectations for isolation, characterisation, contamination control and transparent reporting, making cross-study comparability and reproducibility foundational rather than optional.

## 8. Beyond the Vesicle: The Interplay of the Protein Corona and Non-Vesicular Pathways in Intercellular Signalling

EVs are frequently proposed as specialised mediators of intercellular communication. However, such claims require validation against other established pathways, including soluble factors, direct cell–cell contact, and tunnelling nanotubes (TNTs). Without appropriate controls, effects may be incorrectly attributed to EVs.

A central challenge is the accurate assignment of function to EV-associated cargo. Even refined isolates contain non-vesicular components and peripherally associated proteins. Density gradient profiling indicates that most proteins recovered with small EV fractions are membrane-associated, with only a limited subset consistent with soluble luminal content [113]. Additionally, EVs acquire a biomolecular corona in biofluids that can alter their biological identity. Direct imaging of serum-derived EVs confirms the presence of a native corona, which can be modified or removed during isolation, potentially confounding functional interpretation [114]. Because corona removal may also disrupt vesicles, orthogonal methods such as microfluidic resistive pulse sensing are necessary to distinguish surface clearing from vesicle damage [115]. Surface functionalisation does not bypass this issue; uncontrolled protein adsorption can dominate the engineered surface chemistry and compromise targeting [116].

Conceptually, EV-mediated transfer overlaps with contact-dependent mechanisms. Membrane protrusions such as filopodia and cytonemes can facilitate EV release and are functionally linked to TNT-like structures, suggesting a continuum of intercellular transport [117]. TNTs enable direct exchange of organelles and regulatory RNAs, contributing to tumour adaptation and therapy resistance [118,119]. Together, soluble factors, TNTs, and EVs represent parallel communication systems with unresolved interdependencies [120].

Thus, EV research should adopt a comparative framework. Functional studies must include controls for soluble mediators (including corona-forming proteins) and direct intercellular connections to establish causal specificity and avoid artefactual conclusions.

## 9. Extracellular Vesicles as Therapeutic Vehicles: Clinical Use Cases, Manufacturing Constraints, and Outlook

EVs are being developed as medicines within two complementary clinical paradigms. First, EVs are investigated as biological therapeutics that transfer a complex molecular signal set from producer cells to injured tissues to modulate inflammation and repair processes in a cell-free format [120,121,122,123,124,125]. This rationale supports early-phase trials for inflammatory conditions like acute respiratory distress syndrome (ARDS) and COVID-19 using intravenous or inhaled delivery [123,124]. Second, EVs are engineered as delivery vehicles for active substances, such as nucleic acids, when conventional formulations fail to achieve sufficient target exposure or must cross restrictive biological barriers [121,122,124,125,126]. This approach is being explored in neurological disorders, where intranasal administration aims to enhance central nervous system exposure while limiting systemic clearance [123,124].

Oncology exemplifies both concepts and the necessity for precision engineering. EVs can be functionalised for selective delivery or designed to modulate the tumour microenvironment, addressing immune evasion and therapy resistance that single-agent approaches often miss [127]. Clinical feasibility is demonstrated by dendritic cell-derived EV vaccines evaluated in Phase II for non-small cell lung cancer, and by mesenchymal stromal cell-derived EVs loaded with KRASG12D siRNA for pancreatic cancer in Phase I [126]. Thus, EVs are framed not merely as carriers but as multi-target platforms that address both tumour cells and their microenvironment [127].

The primary advantages proposed for EV therapeutics are biological compatibility, natural membrane architecture facilitating cellular uptake, and engineering flexibility [121,122,127,128]. They enable local delivery paradigms, such as inhalation or intranasal administration, to increase site-specific contact [123,124,126]. However, significant limitations persist. EV preparations are inherently heterogeneous, with composition and drug loading sensitive to cell source and culture conditions, complicating dose definition and comparability [122,124,126,129]. Upon systemic administration, rapid redistribution to the mononuclear phagocyte system limits bioavailability at the target site, making route selection disease-specific [124,126,130]. Furthermore, claims about tissue targeting can be distorted by tracking methods like lipophilic dyes, which may generate artefacts [130]. Manufacturing and standardisation remain the dominant barriers, with common methods like differential ultracentrifugation facing scalability and purity challenges that GMP workflows must resolve [122,124,126,129].

Future perspectives are therefore pragmatic and translational. Progress will depend on scalable production, defined release criteria, stability control, and validated potency assays linking measurable attributes to reproducible effects in humans [121,122,129,131]. Engineering priorities include improving target tissue uptake, enhancing intracellular delivery, and reducing batch variability through better process control [127,128,131]. If these challenges are solved, EV therapeutics are positioned to mature into a distinct drug class that complements existing modalities, particularly where barrier penetration or microenvironment modulation is critical for efficacy [121,122,131].

## 10. Summary

The manuscript examines the history of extracellular vesicle research not as a narrative supplement but as methodological evidence that biological interpretation requires rigorous entity definition. Wolf’s platelet dust experiments demonstrated procoagulant activity in a sedimentable particulate fraction using PF3 assays and electron microscopy, yet by current standards lacked preanalytical controls, marker-based profiling, and the capacity to distinguish tissue factor from phosphatidylserine-driven effects. Crawford’s 1971 [44] work strengthened the membrane particle inference through combined platelet-free plasma preparation, differential centrifugation, ultrastructural visualisation, and biochemical assays, but predated orthogonal purification and subpopulation phenotyping, leaving co-sedimenting non-vesicular material and compositional heterogeneity unresolved.

Contemporary EV research faces structural limitations rather than mere checklist compliance issues. MISEV cannot function as a definitive measurement framework because the underlying problem remains underdetermined and universal subtype markers are indefensible. Nomenclature inflation around exosomes facilitates overclaiming, while EV TRACK data document only modest reporting improvements following guideline introduction. The translational interface exhibits auditability failures, with clinical trial records frequently omitting isolation and characterisation methodologies.

Future directions require methodological deliverables rather than aspirational themes. Metrology must deliver harmonised contaminant-aware EV quantification with explicit reporting of size distributions, adsorptive losses, and lipoprotein co-isolation to enable cross-study comparability. Mechanistic progress demands single-particle and rare-subset resolution because functional capacity varies by cellular origin even within marker defined subsets and isolation methodology alters EV properties and recipient cell effects. Therapeutic development requires scalable manufacturing, defined release criteria, stability controls, and validated potency assays linking measurable attributes to reproducible human effects. Biodistribution constraints include rapid mononuclear phagocyte system uptake, route-dependent variability, and lipophilic dye artefacts, making targeting, intracellular delivery, and process control the engineering priorities for reducing batch variability and enabling interpretable clinical claims.

## Figures and Tables

**Table 1:** ISEV annual meetings: a chronological list of conference locations and the temporary shift to an online format during the pandemic period [84].

Year	Location
ISEV2012	Gothenburg, Sweden
ISEV2013	Boston, United States of America
ISEV2014	Rotterdam, the Netherlands
ISEV2015	Washington, United States of America
ISEV2016	Rotterdam, the Netherlands
ISEV2017	Toronto, Canada
ISEV2018	Barcelona, Spain
ISEV2019	Kyoto, Japan
ISEV2020	Virtual meeting
ISEV2021	Virtual meeting
ISEV2022	Lyon, France
ISEV2023	Seattle, United States of America
ISEV2024	Melbourne, Australia
ISEV2025	Vienna, Austria

Abbreviations: ISEV, International Society for Extracellular Vesicles.

## Data Availability

No new data were created or analysed in this study.

## References

[1] Vickers K.C., Palmisano B.T., Shoucri B.M., Shamburek R.D., Remaley A.T. (2011). MicroRNAs are transported in plasma and delivered to recipient cells by high-density lipoproteins. Nat. Cell Biol..

[2] Jeppesen D.K., Frank A.C., Gammelgaard O.L., Howard K.A. (2023). Extracellular vesicles and nanoparticles: Emerging complexities. Trends Cell Biol..

[3] Kalluri R. (2024). The biology and function of extracellular vesicles in immune response and immunity. Immunity.

[4] van der Pol E., Böing A.N., Gool E.L., Nieuwland R. (2012). Classification, functions, and clinical relevance of extracellular vesicles. Pharmacol. Rev..

[5] Gould S.J., Raposo G. (2013). As we wait: Coping with an imperfect nomenclature for extracellular vesicles. J. Extracell. Vesicles.

[6] Ruzycka-Ayoush M., Prochorec-Sobieszek M., Cieszanowski A., Glogowski M., Szumera-Cieckiewicz A., Podgorska J., Targonska A., Sobczak K., Mosieniak G., Grudzinski I.P. (2024). Extracellular Vesicles as Next-Generation Biomarkers in Lung Cancer Patients: A Case Report on Adenocarcinoma and Squamous Cell Carcinoma. Life.

[7] Raposo G., Stoorvogel A. (2021). Extracellular vesicles: A new perspective on cell-cell communication. Nat. Rev. Mol. Cell Biol..

[8] van Niel G., D’Angelo G., Raposo G. (2018). Shedding light on the cell biology of extracellular vesicles. Nat. Rev. Mol. Cell Biol..

[9] Kalluri A., LeBleu W. (2020). Emerging roles of extracellular vesicles in cancer. Nat. Rev. Cancer.

[10] Wang Y. (2021). Extracellular vesicles and their emerging role in cardiovascular diseases. Cardiovasc. Res..

[11] Yáñez-Mó M., Siljander P.R.-M., Andreu Z., Zavec A.B., Borràs F.E., Buzas E.I., Buzas K., Casal E., Cappello F., Carvalho J. (2015). Biological properties of extracellular vesicles and their physiological functions. J. Extracell. Vesicles.

[12] Yuan Q., Li X.-D., Zhang S.-M., Wang H.-W., Wang Y.-L. (2021). Extracellular vesicles in neurodegenerative diseases: Insights and new perspectives. Genes Dis..

[13] Turpin D., Truchetet M.E., Faustin B., Augusto J.F., Contin-Bordes C., Brisson A., Blanco P., Duffau P. (2016). Role of extracellular vesicles in autoimmune diseases. Autoimmun. Rev..

[14] Cheung K.H., Keerthikumar S., Roncaglia P., Subramanian S.L., Roth M.E., Samuel M., Anand S., Gangoda L., Gould S., Alexander R. (2016). Extending gene ontology in the context of extracellular RNA and vesicle communication. J. Biomed. Semant..

[15] Fusco C., De Rosa G., Spatocco I., Vitiello E., Procaccini C., Frigè C., Pellegrini V., La Grotta R., Furlan R., Matarese G. (2024). Extracellular vesicles as human therapeutics: A scoping review of the literature. J. Extracell. Vesicles..

[16] Du S., Li X., Li Y., Li Q., Zhao Y., Li M., Li X., Ding J., Zhou X., Huang Y. (2023). Extracellular vesicles: A rising star for therapeutics and drug delivery. J. Nanobiotechnol..

[17] Su X., Wang H., Li Q., Chen Z. (2025). Extracellular vesicles: A review of their therapeutic potentials, sources, biodistribution, and administration routes. Int. J. Nanomed..

[18] Van Delen M., Derdelinckx J., Wouters K., Nelissen I., Cools N. (2024). A systematic review and meta-analysis of clinical trials assessing safety and efficacy of human extracellular vesicle-based therapy. J. Extracell. Vesicles.

[19] PubMed (National Library of Medicine) Search Query for Extracellular Vesicle-Related Publications Published Since 2010. https://pubmed.ncbi.nlm.nih.gov.

[20] Guo S., Huang J., Li G., Chen W., Li Z., Lei J. (2023). The role of extracellular vesicles in circulating tumor cell-mediated distant metastasis. Mol. Cancer.

[21] Bebelman M.P., Smit M.J., Pegtel D.M., Baglio S.R. (2018). Biogenesis and function of extracellular vesicles in cancer. Pharmacol. Ther..

[22] Skog J., Würdinger T., van Rijn S., Meijer D.H., Gainche L., Sena-Esteves M., Curry W.T., Carter B.S., Krichevsky A.M., Breakefield X.O. (2008). Glioblastoma microvesicles transport RNA and proteins that promote tumour growth and provide diagnostic biomarkers. Nat. Cell Biol..

[23] Nowak M., Górczyńska J., Kołodzińska K., Rubin J., Choromańska A. (2023). Extracellular vesicles as drug transporters. Int. J. Mol. Sci..

[24] Wang Y., Cheng L., Zhao H., Li Z., Chen J., Cen Y., Zhang Z. (2022). The therapeutic role of ADSC-EVs in skin regeneration. Front. Med..

[25] Lehmann T.P., Golik M., Olejnik J., Łukaszewska M., Markowska D., Drożdżyńska M., Kotecki A., Głowacki M. (2023). Potential applications of using tissue-specific EVs in targeted therapy and vaccinology. Biomed. Pharmacother..

[26] Lötvall J., Hill A.F., Hochberg F., Buzás E.I., Di Vizio D., Gardiner C., Gho Y.S., Kurochkin I.V., Mathivanan S., Quesenberry P. (2014). Minimal experimental requirements for definition of extracellular vesicles and their functions: A position statement from the International Society for Extracellular Vesicles. J. Extracell. Vesicles.

[27] Hargett L.A., Bauer N.N. (2013). On the origin of microparticles: From “platelet dust” to mediators of intercellular communication. Pulm. Circ..

[28] Cohen S.S. (2010). Erwin Chargaff (1905–2002). A Biographical Memoir, with selected bibliography by Robert Lehman. Biographical Memoirs of the National Academy of Sciences.

[29] Hargittai I. (2000). Candid Science: Conversations With Famous Chemists.

[30] Chargaff E., West R. (1946). The biological significance of the thromboplastic protein of blood. J. Biol. Chem..

[31] Chargaff E., Nord W.F., Weakmen C.H. (1945). The coagulation of blood. Advances in Enzymology and Related Areas of Molecular Biology.

[32] Chargaff E. (1942). The phospholipid components of blood coagulation. J. Biol. Chem..

[33] West R. (1948). Activity of vitamin B12 in Addisonian pernicious anemia. Science.

[34] West R. (1927). On feeding certain liver constituents in pernicious anemia. Proc. Soc. Exp. Biol. Med..

[35] West R. (1929). Pernicious anemia as a deficiency disease. Ann. Intern. Med..

[36] West R., Nichols E.G. (1928). Liver fractions in pernicious anemia. JAMA.

[37] Obituary: Randolph West. Columbia University Medical Center Library Archives. https://www.library-archives.cumc.columbia.edu/obit/west-randolph.

[38] (1949). The Joseph Goldberger Award in Nutrition. JAMA.

[39] American Chemical Society National Historic Chemical Landmarks: The Vitamin B Complex. https://www.acs.org/education/whatischemistry/landmarks/vitamin-b-complex.html.

[40] West R., Reisner E.H. (1949). Treatment of pernicious anemia with crystalline vitamin B12. Am. J. Med..

[41] Wolf P. (1967). The nature and significance of platelet products in human plasma. Br. J. Haematol..

[42] O’Brien J.R. (1955). The platelet-like activity of serum. Br. J. Haematol..

[43] Hougie C. (1955). The activation of platelets by plasma. Br. J. Haematol..

[44] Crawford N. (1971). The presence of contractile proteins in platelet microparticles isolated from human and animal platelet-free plasma. Br. J. Haematol..

[45] Johnstone R.M. (2005). Revisiting the road to the discovery of exosomes. Blood Cells Mol. Dis..

[46] The Canadian Encyclopedia. Rose Johnstone. https://www.thecanadianencyclopedia.ca/en/article/rose-johnstone.

[47] McGill University Department of Biochemistry In Memoriam: Rose Johnstone. https://www.mcgill.ca/biochemistry/about-us/memoriam.

[48] Johnstone R.M., Gillett M., Sibblad K. (1984). Feeling outside on the inside. A Fair Shake: Autobiographical Essays by McGill Women.

[49] West Liberty University Philip Stahl–Wall of Honor. https://westliberty.edu/alumni/wall-of-honor/name/philip-stahl/.

[50] Washington University School of Medicine Stahl Honored by Research Society. https://medicine.wustl.edu/news/stahl-honored-research-society.

[51] Bradshaw R.A., Stahl P.D., Hart G.W. (2022). Encyclopedia of Cell Biology.

[52] Harding C., Heuser J., Stahl P. (1983). Receptor-mediated endocytosis of transferrin and recycling of the transferrin receptor in rat reticulocytes. J. Cell Biol..

[53] Pan B.T., Johnstone R.M. (1983). Fate of the transferrin receptor during maturation of sheep reticulocytes in vitro: Selective externalization of the receptor. Cell.

[54] Johnstone R.M., Adam M., Hammond J.R., Orr L., Turbide C. (1987). Vesicle formation during reticulocyte maturation: Association of plasma membrane activities with released vesicles (exosomes). J. Biol. Chem..

[55] Witwer K.W., Théry C. (2019). Extracellular vesicles or exosomes? On primacy, precision, and popularity influencing a choice of nomenclature. J. Extracell. Vesicles.

[56] Johnstone R.M., Mathew A., Mason A.B., Teng K. (1991). Exosome formation during maturation of mammalian and avian reticulocytes: Evidence that exosome release is a major route for externalization of obsolete membrane proteins. J. Cell Physiol..

[57] Raposo G., Nijman H.W., Stoorvogel W., Liejendekker R., Harding C.V., Melief C.J., Geuze H.J. (1996). B lymphocytes secrete antigen-presenting vesicles. J. Exp. Med..

[58] Institut Curie Maria da Graça Benedetti Raposo. https://institut-curie.org/person/maria-da-graca-benedetti-raposo.

[59] Zitvogel L., Regnault A., Lozier A., Wolfers J., Flament C., Tenza D., Ricciardi-Castagnoli P., Raposo G., Amigorena S. (1998). Eradication of established murine tumors using a novel cell-free vaccine: Dendritic cell-derived exosomes. Nat. Med..

[60] Ratajczak J., Miekus K., Kucia M., Zhang J., Reca R., Dvorak P., Ratajczak M.Z. (2006). Embryonic stem cell-derived microvesicles reprogram hematopoietic progenitors: Evidence for horizontal transfer of mRNA and protein delivery. Leukemia.

[61] Ratajczak J., Wysoczynski M., Hayek F., Janowska-Wieczorek A., Ratajczak M.Z. (2006). Membrane-derived microvesicles: Important and underappreciated mediators of cell-to-cell communication. Leukemia.

[62] Ratajczak M.Z., Zuba-Surma E., Wojakowski W., Suszynska M., Mierzejewska K., Liu R., Ratajczak J., Shin D.M., Kucia M. (2014). Very small embryonic-like stem cells (VSELs) represent a real challenge in stem cell biology: Recent pros and cons in the midst of a lively debate. Leukemia.

[63] Kucia M., Reca R., Campbell F.R., Zuba-Surma E., Majka M., Ratajczak J., Ratajczak M.Z. (2006). A population of very small embryonic-like (VSEL) CXCR4+SSEA-1+Oct-4+ stem cells identified in adult bone marrow. Leukemia.

[64] Ratajczak J., Miekus K., Kucia M., Zhang J., Reca R., Dvorak P., Ratajczak M.Z. (2006). Embryonic stem cell-derived microvesicles induce phenotypic reprogramming of hematopoietic progenitors. Stem Cells Dev..

[65] Libura J., Drukala J., Majka M., Tomescu O., Navenot J.M., Kucia M., Marquez L., Peiper S.C., Barr F.G., Janowska-Wieczorek A. (2002). CXCR4-SDF-1 signaling is active in rhabdomyosarcoma cells and regulates locomotion, chemotaxis and adhesion. Blood.

[66] Miyanishi M., Mori Y., Seita J., Chen J.Y., Karten S., Chan C.K., Nakauchi H., Weissman I.L. (2013). Do pluripotent stem cells exist in adult mice as very small embryonic stem cells?. Stem Cell Rep..

[67] Lai C.P., Kim E.Y., Badr C.E., Weissleder R., Mempel T.R., Tannous B.A., Breakefield X.O. (2015). Visualization and tracking of tumour extracellular vesicle delivery and RNA translation using multiplexed reporters. Nat. Commun..

[68] Piffoux M., Volatron J., Silva A.K.A., Gazeau F. (2021). Thinking quantitatively of RNA-based information transfer via extracellular vesicles: Lessons to learn for the design of RNA-loaded EVs. Pharmaceutics.

[69] O’Brien K., Breyne K., Ughetto S., Laurent L.C., Breakefield X.O. (2020). RNA delivery by extracellular vesicles in mammalian cells and its applications. Nat. Rev. Mol. Cell Biol..

[70] Mathieu M., Martin-Jaular L., Lavieu G., Théry C. (2019). Specificities of secretion and uptake of exosomes and other extracellular vesicles for cell-to-cell communication. Nat. Cell Biol..

[71] Théry C., Witwer K.W., Aikawa E., Alcaraz M.J., Anderson J.D., Andriantsitohaina R., Antoniou A., Arab T., Archer F., Atkin-Smith G.K. (2018). Minimal information for studies of extracellular vesicles 2018 (MISEV2018): A position statement of the International Society for Extracellular Vesicles and update of the MISEV2014 guidelines. J. Extracell. Vesicles.

[72] Ratajczak M.Z., Thetchinamoorthy K., Wierzbicka D., Konopko A., Ratajczak J., Kucia M. (2025). Extracellular microvesicles/exosomes—Magic bullets in horizontal transfer between cells of mitochondria and molecules regulating mitochondria activity. Stem Cells.

[73] Kalluri R., LeBleu V.S. (2020). The biology, function, and biomedical applications of exosomes. Science.

[74] Nunez E.A., Wallis J., Gershon M.D. (1974). Secretory processes in follicular cells of the bat thyroid. III. the occurrence of extracellular vesicles and colloid droplets during arousal from hibernation. Am. J. Anat..

[75] Halperin W., Jensen W.A. (1967). Ultrastructural changes during growth and embryogenesis in carrot cell cultures. J. Ultrastruct. Res..

[76] Regente M., Corti-Monzón G., Maldonado A.M., Pinedo M., Jorrín J., de la Canal L. (2009). Vesicular fractions of sunflower apoplastic fluids are associated with potential exosome marker proteins. FEBS Lett..

[77] Rodrigues M.L., Nakayasu E.S., Oliveira D.L., Nimrichter L., Nosanchuk J.D., Almeida I.C., Casadevall A. (2008). Extracellular vesicles produced by Cryptococcus neoformans contain protein components associated with virulence. Eukaryot. Cell.

[78] Anderson H.C. (1969). Vesicles associated with calcification in the matrix of epiphyseal cartilage. J. Cell Biol..

[79] Wolf P. (1967). Platelet-derived material in plasma: Significance in coagulation studies. Thromb. Diath. Haemorrh..

[80] Valadi H., Ekström K., Bossios A., Sjöstrand M., Lee J.J., Lötvall J.O. (2007). Exosome-mediated transfer of mRNAs and microRNAs is a novel mechanism of genetic exchange between cells. Nat. Cell Biol..

[81] Mathivanan S., Lim J.W., Tauro B.J., Ji H., Moritz R.L., Simpson R.J. (2010). Proteomics analysis of A33 immunoaffinity-purified exosomes released from the human colon tumor cell line LIM1215 reveals a tissue-specific protein signature. Mol. Cell Proteom..

[82] Dragovic R.A., Gardiner C., Brooks A.S., Tannetta D.S., Ferguson D.J.P., Hole P., Carr B., Redman C.W.G., Harris A.L., Dobson P.J. (2011). Sizing and phenotyping of cellular vesicles using nanoparticle tracking analysis. Nanomedicine.

[83] Welsh J.A., Van Der Pol E., Arkesteijn G.J.A., Bremer M., Brisson A., Coumans F., Dignat-George F., Duggan E., Ghiran I., Giebel B. (2020). MIFlowCyt-EV: A framework for standardized reporting of extracellular vesicle flow cytometry experiments. J. Extracell. Vesicles.

[84] Cai Q., Halilovic L., Shi T., Chen A., He B., Wu H., Jin H. (2023). Extracellular vesicles: Cross-organismal RNA trafficking in plants, microbes, and mammalian cells. Extracell. Vesicles Circ. Nucleic Acids.

[85] Liu J.Y., Yu Z.L., Fu Q.Y., Zhang L.Z., Li J.B., Wu M., Liu B., Chen G. (2023). Immunosuppressive effect of small extracellular vesicle PD-L1 is restricted by co-expression of CD80. Br. J. Cancer.

[86] Phan T.H., Umakoshi H., Shimanouchi T., Kuboi R., Yamada H., Kato K., Tsuboi Y., Yamamoto T., Ito Y., Saito T. (2021). New multiscale characterization methodology for effective determination of isolation-structure-function relationship of extracellular vesicles. Front. Bioeng. Biotechnol..

[87] Zeng H., Guo S., Ren X., Wu Z., Liu S., Yao X. (2023). Current strategies for exosome cargo loading and targeting delivery. Cells.

[88] Chen Y., Douanne N., Wu T., Kaur I., Tsering T., Erzingatzian A., Nadeau A., Juncker D., Nerguizian V., Burnier J.V. (2025). Leveraging nature’s nanocarriers: Translating insights from extracellular vesicles to biomimetic synthetic vesicles for biomedical applications. Sci. Adv..

[89] Shami-Shah A., Travis B.G., Walt D.R. (2023). Advances in extracellular vesicle isolation methods: A path towards cell-type specific EV isolation. Extracell. Vesicles Circ. Nucleic Acids.

[90] Kang K., Zhang Y., Zhou X., Yu Y., Zhu N., Cheng J., Yi Q., Wu Y. (2023). Hybrid extracellular vesicles-liposomes camouflaged magnetic vesicles cooperating with bioorthogonal click chemistry for high-efficient melanoma circulating tumor cells enrichment. Adv. Healthc. Mater..

[91] Mizenko R.R., Feaver M., Bozkurt B.T., Lowe N., Nguyen B., Huang K.W., Wang A., Carney R.P. (2024). A critical systematic review of extracellular vesicle clinical trials. J. Extracell. Vesicles.

[92] McKiernan J., Donovan M.J., O’Neill V., Bentink S., Noerholm M., Belzer S., Skog J., Kattan M.W., Partin A.W., Andriole G. (2016). A novel urine exosome gene expression assay to predict high-grade prostate cancer at initial biopsy. JAMA Oncol..

[93] Kalluri V.S., LeBleu V.S., Sugimoto H., Yang J., Choi M.R., Banerjee S., Wang X., Wang H., Lee H., Di Vizio D. (2025). Engineered exosomes with KrasG12D specific siRNA in pancreatic cancer: A phase I study with immunological correlates. Nat. Commun..

[94] Besse B., Charrier M., Lapierre V., Dansin E., Lantz O., Planchard D., Le Chevalier T., Livartoski A., Barlesi F., Laplanche A. (2015). Dendritic cell-derived exosomes as maintenance immunotherapy after first line chemotherapy in NSCLC. Oncoimmunology.

[95] Busatto S., Vilanilam G., Ticer T., Lin W.L., Dickson D.W., Shapiro S., Bergese P., Wolfram J. (2018). Tangential flow filtration for highly efficient concentration of extracellular vesicles from large volumes of fluid. Cells.

[96] Casajuana Ester M., Day R.M. (2023). Production and utility of extracellular vesicles with 3D culture methods. Pharmaceutics.

[97] Yang Z., Shi J., Xie J., Wang Y., Sun J., Liu T., Zhao Y., Zhao X., Wang X., Ma Y. (2020). Large-scale generation of functional mRNA-encapsulating exosomes via cellular nanoporation. Nat. Biomed. Eng..

[98] Welsh J.A., Théry C., Hendrix A., Aikawa E., Archbold H.C., Bacigalupo M.L., Buzás E.I., Carter D.R.F., Clayton A., Coumans F.A.W. (2024). Minimal information for studies of extracellular vesicles (MISEV2023): From basic to advanced approaches. J. Extracell. Vesicles.

[99] Witwer K.W., Soekmadji C., Hill A.F., Wauben M.H.M., Buzás E.I., Di Vizio D., Falcon-Perez J.M., Gardiner C., Hochberg F.H., Kurochkin I.V. (2017). Updating the MISEV minimal requirements for extracellular vesicle studies: Building bridges to reproducibility. J. Extracell. Vesicles.

[100] Witwer K.W., Théry C., Buzás E.I., Di Vizio D., Falcon-Perez J.M., Gardiner C., Hill A.F., Hochberg F.H., Kurochkin I.V., Lötvall J. (2021). Updating MISEV: Evolving the minimal requirements for studies of extracellular vesicles. J. Extracell. Vesicles.

[101] Lötvall J., Rajendran L., Gho Y.S., Thery C., Wauben M., Raposo G., Sjöstrand M., Taylor D., Telemo E., Breakefield X.O. (2012). The launch of Journal of Extracellular Vesicles (JEV), the official journal of the International Society for Extracellular Vesicles–about microvesicles, exosomes, ectosomes and other extracellular vesicles. J. Extracell. Vesicles.

[102] International Society for Extracellular Vesicles Journal of Extracellular Vesicles. https://www.isev.org/journal-of-extracellular-vesicles.

[103] Akbar N., Théry C., Witwer K.W., Hill A.F., Lötvall J., Buzás E.I., Gardiner C., Hendrix A., Di Vizio D., Falcon-Perez J.M. (2026). Updating MISEV: When, what, how, and why?. J. Extracell. Vesicles.

[104] Sharma M., Lozano-Amado D., Chowdhury D., Singh U. (2023). Extracellular vesicles and their impact on the biology of protozoan parasites. Trop. Med. Infect. Dis..

[105] Van Deun J., Hendrix A., On behalf of the EV-TRACK consortium (2017). Is your article EV-TRACKed?. J. Extracell. Vesicles.

[106] Wiley Online Library Journal Metrics–Journal of Extracellular Vesicles. https://isevjournals.onlinelibrary.wiley.com/journal/20013078/journal-metrics.

[107] International Society for Extracellular Vesicles Journal of Extracellular Biology. https://www.isev.org/journal-of-extracellular-biology.

[108] International Society for Extracellular Vesicles About ISEV. https://www.isev.org/about-isev.

[109] International Society for Extracellular Vesicles (ISEV) Past Annual Meetings. https://www.isev.org/past-annual-meetings.

[110] EV-TRACK Consortium, Van Deun J., Mestdagh P., Agostinis P., Akay Ö., Anand S., Anckaert J., Martinez Z.A., Baetens T., Beghein E. (2017). EV-TRACK: Transparent reporting and centralizing knowledge in extracellular vesicle research. Nat. Methods.

[111] Kalra H., Simpson R.J., Ji H., Aikawa E., Altevogt P., Askenase P., Bond V.C., Borràs F.E., Breakefield X., Budnik V. (2012). Vesiclepedia: A compendium for extracellular vesicles with continuous community anno-tation. PLoS Biol..

[112] Keerthikumar S., Chisanga D., Ariyaratne D., Al Saffar H., Anand S., Zhao K., Samuel M., Pathan M., Jois M., Chilamkurti N. (2016). ExoCarta: A web-based compendium of exosomal cargo. J. Mol. Biol..

[113] Morales-Sanfrutos J., Etxeberria-Ugartemendia J., Barroso-Gomila O., González E., Sendino M., Ximénez-Embún P., García F., Zarzuela E., Falcón-Pérez J.M., Peinado H. (2026). Defining the reference proteomes for small extracellular vesicles and non-vesicular components. Nat. Cell Biol..

[114] Poletaeva J.E., Tupitsyna A.V., Grigor’eva A.E., Dovydenko I.S., Ryabchikova E.I. (2024). Attempts to preserve and visualize protein corona on the surface of biological nanoparticles in blood serum using photomodification. Beilstein J. Nanotechnol..

[115] Sonallya Tasvilla Gaál A., Szigyarto I., Varga Z. (2025). Biophysical profiling of protein corona on red blood cell-derived extracellular vesicles (REVs): Linear dichroism and microfluidic resistive pulse sensing separate surface clearing from vesicle disruption. Methods Mol. Biol..

[116] Musicò A., Zenatelli R., Romano M., Zendrini A., Alacqua S., Tassoni S., Paolini L., Urbinati C., Rusnati M., Bergese P. (2023). Surface functionalization of extracellular vesicle nanoparticles with antibodies: A first study on the protein corona “variable”. Nanoscale Adv..

[117] Hu H.T., Nishimura T., Kawana H., Dante R.A.S., D’Angelo G., Suetsugu S. (2024). The cellular protrusions for inter-cellular material transfer: Similarities between filopodia, cytonemes, tunneling nanotubes, viruses, and extracellular vesicles. Front. Cell Dev. Biol..

[118] Lou E., Zhai E., Sarkari A., Desir S., Wong P., Iizuka Y., Yang J., Subramanian S., McCarthy J., Bazzaro M. (2018). Cellular and molecular networking within the ecosystem of cancer cell communication via tunneling nanotubes. Front. Cell Dev. Biol..

[119] Lou E. (2020). A ticket to ride: The implications of direct intercellular communication via tunneling nanotubes in peritoneal and other invasive malignancies. Front. Oncol..

[120] Ariazi J., Benowitz A., De Biasi V., Den Boer M.L., Cherqui S., Cui H., Douillet N., Eugenin E.A., Favre D., Goodman S. (2017). Tunneling nanotubes and gap junctions—Their role in long-range intercellular communication during development, health, and disease conditions. Front. Mol. Neurosci..

[121] György B., Hung M.E., Breakefield X.O., Leonard J.N. (2015). Therapeutic applications of extracellular vesicles: Clinical promise and open questions. Annu. Rev. Pharmacol. Toxicol..

[122] Wiklander O.P.B., Brennan M.Á., Lötvall J., Breakefield X.O., El Andaloussi S. (2019). Advances in therapeutic applications of extracellular vesicles. Sci. Transl. Med..

[123] Sanz-Ros J., Mas-Bargues C., Romero-García N., Huete-Acevedo J., Dromant M., Borrás C. (2023). Extracellular vesicles as therapeutic resources in the clinical environment. Int. J. Mol. Sci..

[124] Klyachko N.L., Arzt C.J., Li S.M., Gololobova O.A., Batrakova E.V. (2020). Extracellular vesicle-based therapeutics: Preclinical and clinical investigations. Pharmaceutics.

[125] Kumar M.A., Ranjan R., De A., Jha A.K., Pathak P., Mishra S., Sahoo A., Jha N.K., Yadav S.K., Kumar D. (2024). Extracellular vesicles as tools and targets in therapy for diseases. Signal Transduct. Target. Ther..

[126] Hu M., Han Y., Zhang X., Tian S., Shang Z., Yuan Z., He L. (2025). Extracellular vesicles for targeted drug delivery: Advances in surface modification strategies and therapeutic applications. J. Transl. Med..

[127] Ghosal S., Gupta S., Das S., Chakrabarti S., Mandal P., Bandyopadhyay S., Bhattacharya S., Dutta S., Mukherjee S., Chatterjee M. (2025). Revolutionizing therapeutics: Unleashing the power of extracellular vesicles for disease intervention. Curr. Opin. Physiol..

[128] Esmaeili A., Alini M., Baghaban Eslaminejad M., Hosseini S. (2022). Engineering strategies for customizing extracellular vesicle uptake in a therapeutic context. Stem Cell Res. Ther..

[129] Du X., Yang Y., Li Q., Zhang H., Wang J., Liu Y., Zhao Y., Chen L., Sun W., Zhou Y. (2025). Extracellular vesicles as precision therapeutic vectors: Charting the future of cell-targeted therapies. Precis. Med. Eng..

[130] Di Rocco G., Baldari S., Toietta G. (2016). Towards therapeutic delivery of extracellular vesicles: Strategies for in vivo tracking and biodistribution analysis. Stem Cells Int..

[131] Ilamathi H.S., Rinaldo G., Wiklander O.P.B. (2025). Advances in extracellular vesicle-based cancer precision medicine. Cancer Lett..

